# Protection by Neuroglobin Expression in Brain Pathologies

**DOI:** 10.3389/fneur.2016.00146

**Published:** 2016-09-12

**Authors:** Eliana Baez, Valentina Echeverria, Ricardo Cabezas, Marco Ávila-Rodriguez, Luis Miguel Garcia-Segura, George E. Barreto

**Affiliations:** ^1^Departamento de Nutrición y Bioquimica, Facultad de Ciencias, Pontificia Universidad Javeriana, Bogotá D.C., Colombia; ^2^Facultad de Ciencias de la Salud, Universidad San Sebastián, Concepción, Chile; ^3^Instituto Cajal, CSIC, Madrid, Spain; ^4^Instituto de Ciencias Biomédicas, Universidad Autónoma de Chile, Santiago, Chile

**Keywords:** astrocytes, neuroglobin, mitochondria, neuroprotection, brain injury

## Abstract

Astrocytes play an important role in physiological, metabolic, and structural functions, and when impaired, they can be involved in various pathologies including Alzheimer, focal ischemic stroke, and traumatic brain injury. These disorders involve an imbalance in the blood flow and nutrients such as glucose and lactate, leading to biochemical and molecular changes that cause neuronal damage, which is followed by loss of cognitive and motor functions. Previous studies have shown that astrocytes are more resilient than neurons during brain insults as a consequence of their more effective antioxidant systems, transporters, and enzymes, which made them less susceptible to excitotoxicity. In addition, astrocytes synthesize and release different protective molecules for neurons, including neuroglobin, a member of the globin family of proteins. After brain injury, neuroglobin expression is induced in astrocytes. Since neuroglobin promotes neuronal survival, its increased expression in astrocytes after brain injury may represent an endogenous neuroprotective mechanism. Here, we review the role of neuroglobin in the central nervous system, its relationship with different pathologies, and the role of different factors that regulate its expression in astrocytes.

## Introduction

The brain has about 170 billion cells (1), which consume an average of 516 kcal of energy per day, representing 22% of total energy demand of an organism (2). This energy demand is required to carry out essential functions such as synaptic transmission, uptake and metabolism of neurotransmitters, and maintenance of ion gradients (3). For this reason, it is of pivotal importance to maintain optimal conditions of the intra- and extracellular environment targeting nerve cells needs. However, in diseases such as ischemic and traumatic brain injuries, an energy imbalance induced by the interruption of blood flow leads to metabolic stress, ionic disturbance, and activation of a complex cascade of biochemical and molecular events that can cause neuronal death (4). Moreover, there are other diseases such as hypoglycemia and diabetes, in which a misbalance in glucose levels can trigger brain damage (5, 6). In this context, traumatic brain injury has become a global public health problem, and it is the leading cause of death in individuals under 45 years of age and recurrent in young people, adolescents, and elders (7). Brain trauma induces cognitive and motor dysfunction (8). In a study reported by Quijano et al., cognitive abilities were assessed in subjects who suffered moderate head trauma and a control non-injured group. The results revealed significant differences in orientation, attention, memory, language, reading, and writing abilities (9). Despite the enormous efforts and progress in research, treatment strategies for traumatic brain injury are still limited, and currently, there are no effective treatments against their consequences. It has been described that after injury, the first phase involves focal hematoma and diffuse edema that results in white matter damage. The second phase involves additional pathological cellular and molecular events such as the abnormal release of neurotransmitters, the generation of free radicals, Ca^2+^-signaling abnormalities, apoptotic factors activation, and mitochondrial dysfunction leading to neuronal damage, neuroinflammation, and brain dysfunction (10). Moreover, changes that occur in this second phase trigger the death of neurons and astrocytic reactivity. Therefore, it is necessary to search for therapeutic alternatives to prevent further neurological damage and restore CNS homeostasis upon injury.

Neurons are usually affected to a greater extent during injuries, since they have less antioxidant mechanisms than astrocytes being more affected by increased excitotoxicity than glial cells (11). Astrocytes have an active and critical role in the nervous system under normal and pathological conditions. During brain injury and neurodegenerative conditions, astrocytes participate in the removal of toxic molecules and promote neuronal survival throughout the release of trophic factors and antioxidant molecules (12). For example, astrocytes produce various antioxidant molecules, such as glutathione transferase (GSH), superoxide synthase (SOD), and catalase, providing further antioxidant protection to neurons (13). Also, astrocytes integrate the blood–brain barrier (BBB) (13, 14), thus providing active support in the formation of neural connections and brain activity (15). These cells are key regulators of neuronal energy by providing them with lactate (16–18). For all these reasons, astrocytes are vital to restore brain function after injury. In different neurodegenerative conditions such as ischemia–reperfusion injury, a key role is played by mitochondria in the generation of reactive oxygen species (ROS), dysfunctional energy metabolism, and mitochondria-induced apoptosis (19). Likewise, the disruption of synaptic regulation by astroglia seems to play an important role in neurodegeneration and brain damage (20).

It is considered that a transient or permanent impairment of astrocytic functions may negatively impact neurons during pathological conditions. For this reason, it is important to expand the knowledge about the neuroprotective mechanisms mediated by astrocytes during brain injury to find alternatives to prevent altered responses affecting neuroprotection and recovery (21). In this context, neuroglobin (Ngb), a protein expressed astrocytes and neurons, of the central nervous system (CNS) and the peripheral nervous system (PNS) (22, 23) has often been linked to neuroprotection in different neuropathological conditions (24, 25) through antioxidant and antiapoptotic mechanisms (26, 27). The expression of Ngb is induced in human astrocytes during brain injury, possibly as a neuroprotective mechanism (28). Interestingly, Ngb is expressed in astrocytes and neurons of whales and seals as a mechanism to withstand long periods of hypoxia (29). However, more research is needed to completely address the importance of Ngb protective mechanisms and its relationship with astrocyte functions. In the present review article, we explore the role of Ngb in the CNS, focusing on astrocytes and its relationship with different pathologies.

## Astrocytes and Brain Pathologies

Astrocytes are responsible for glucose uptake and release of lactate to neurons (16, 17, 30), which are involved in memory and cognition, glutamate recycling, and synthesis of antioxidant glutathione (31). Furthermore, astrocytes have a unique cellular structure that allows them to detect any change in the environment and dynamically respond to extracellular changes or metabolic requirements, providing sources of energy from the glucose taken from blood flow (32) or from energy reserves such as glycogen (33). In addition to glutathione, astrocytes have a special antioxidant system that includes glutathione peroxidase, heme oxygenase I, and catalase, which are able to detoxify ROS in the brain (32, 34). Astrocytes are also considered polyfunctional cells because they also contribute to the elimination of glutamate (Glu), the major excitatory neurotransmitter in the CNS (35). Moreover, Bergmann glia from the cerebellum express the EAAT1 and EAAT2 transporters (36). In this respect, the EAAT2 (GLT1) is responsible for 90% of glutamate uptake through the astrocyte endfeet that make direct contact with the synapses (37). However, this mechanism of Glu uptake and transport becomes affected during brain pathologies, and the increased levels of Glu in the extracellular space might induce excitotoxicity and the severity of brain injury (38, 39). Other astrocytic functions include the remodeling of the blood brain barrier (14) and production of growth factors (18, 40, 41), which in turn promote cell repair during episodes of injury. Faced with an insult or injury, astrocytes adopt a reactive metabolic phenotype (16, 17, 42–44). This phenotype has a beneficial effect on the preservation of neural tissue and in the restriction of moderate focal inflammation (17, 45, 46). However, when this response is maintained and generalized, it can become counterproductive because astrocytic efforts are redirected toward defensive and repair tasks at the expense of providing adequate metabolic support to neurons and also by blocking axonal regeneration (47). Despite this controversial harmful role of reactive astrocytes, a recent study indicated that astrocyte scar formation might help axon regeneration by augmenting multiple axon-growth-supporting molecules (48), demonstrating that inhibiting glial scar might reduce axon regrowth and worsen CNS damage.

According to Sofroniew (49–51), reactive astrogliosis covers some key characteristics: (1) molecular, cellular, and functional changes in astrocytes related to the severity of injury in the CNS; (2) changes are regulated by specific context of molecules *via* inter- and intracellular signaling; and (3) astrogliosis can exert an alteration in normal astrocytic activities, which in turn can lead to positive or harmful effects in surrounding cells. Additionally, astrocyte gap junctions can remain open after brain injury (52), allowing the entrance of pro-apoptotic factors and immune cells that exacerbate cellular injury (53).

Astrocytes, as other CNS cells, are affected by decreased levels of ATP. This decrease in ATP levels is associated with two fundamental aspects: (i) decreased cerebral blood flow to the range of 100 g^−1^ (5–8.5 ml min^−1^), which leads to irreversible tissue damage by the small amount of glucose and oxygen available (8), and (ii) increased intracellular calcium that leads to damaging calcium levels in mitochondria (54). These facts suggest the importance of astrocytic protection as a potential therapeutic target for neuroprotection and preservation of CNS functions following injury.

### Mitochondrial Function and Dysfunction in Astrocytes

Mitochondria are essential organelles to sustain life and the physiological function of cells under normal conditions by maintaining energy balance through substrate oxidation, modulation of calcium levels, and redox balance (13, 30, 55). However, these organelles are also the main target of oxidative stress (30) by an imbalance between the production of oxidative molecules, such as hydrogen peroxide (H_2_O_2_), superoxide radical (${\text{O}}_{2}^{-}$), and the hydroxyl radical (OH), and the ability of the cell to defend against these radicals (56). Because there is a close relationship between mitochondrial dysfunction and brain injury, mitochondrial protection has become a main therapeutic strategy for treating multiple neurodegenerative diseases, such as Parkinson’s disease (PD), Alzheimer’s disease (AD), and amyotrophic lateral sclerosis, among others (13, 30, 40, 57–59).

As reviewed by Kubik and Philbert (60), from a total of 12,614 mitochondrial investigations in cells of the nervous system, only 1,214 were directed to astrocytic mitochondria. This fact overshadows that mitochondria in astrocytes provide the metabolic substrates necessary for neural function and are essential to maintain the energetic balance of the brain and the production of antioxidants (61, 62). For example, according to Voloboueva et al. (63), the inhibition of mitochondria during glucose deprivation conditions induces functional changes in astrocytes related to a decrease in ATP levels, depolarization of the plasma membrane, and reduced glutamate uptake, without a significant loss of their viability. Therefore, this evidence strongly suggests that the damage to the astrocytic mitochondrial function may be the start of brain lesions and neuronal death (61).

Studies in animal models of PD showed that the administration of 1 and 10 μg of either vascular endothelial growth factor (VEGF) or glial-derived neurotrophic factor (GDNF) increased the expression of mitochondrial genes (64), suggesting that these growth factors may have a role in mitochondrial protection. Similarly, *in vitro* administration of platelet-derived growth factor BB (PDGF-BB) preserved mitochondrial function in astrocytes treated with rotenone (40). Furthermore, another study reported that the transmembrane protein TrkB (a receptor for BDNF) was co-localized with mitochondria in astrocytes (63), suggesting that astrocytes’ mitochondria have the potential to directly interact with neurotrophic factors and other protective proteins such as Ngb (65, 66). Finally, other substances studied in mitochondrial protection are CoQ10 (Coenzyme Q10) and conditioned medium from mesenchymal stem cells (67–69). CoQ10 is a ubiquinone with multiple functions such as decreasing the production of ROS, stabilizing mitochondrial membrane potential, improving mitochondrial respiration, inhibiting mitochondria-mediated pathway of cell death, and activating the mitochondrial biogenesis (69). On the other hand, conditioned medium from mesenchymal stem cells has been shown as a protective substance, which contains proangiogenic and antiapoptotic factors, immunomodulators, antioxidants, and neuronal differentiation factors among others, that improve the mitochondrial protection during injuries (68). However, further studies are necessary in order to find new methodologies for the protection of astrocytic mitochondria.

## Neuroglobin in Brain Pathologies

Oxygen depletion is one of the more detrimental conditions for the CNS, inducing irreversible damage and as a result loss of cognitive functions. Oxygen depletion is underlying several CNS diseases such as ischemia or TBI.

As stated above, aquatic mammals such as whales and seals (29) withstand conditions of severe hypoxia without damage; they are unique models to investigate neuroprotective mechanisms. The comparison of Ngb protein sequence between terrestrial and aquatic mammals revealed minor differences in its sequence of only two or three amino acids, which did not give rise to confer functional differences between both groups. However, Ngb mRNA expression levels were 4–15 times higher in the brain of seals and whales than in those from terrestrial mammals, suggesting that higher Ngb levels in aquatic mammals can be a neuroprotective mechanism against brain hypoxia and ROS production (70). Similarly, in a behavioral study in transgenic mice overexpressing Ngb under normoxia and hypoxia, it was shown that Ngb promotes survival *in vivo* and may play an important role in countering the adverse effects of a hypoxic ischemic stroke. No significant behavioral differences were detected between control and Ngb overexpressing mice at 3 months of age, but transgenic mice showed a superior behavioral performance than control mice at 1 year of age (71).

Traumatic brain injury is another major pathology of the brain, which affects world population. Basic features include bleeding, cell death, increased production of β-amyloid, basic fibroblast growth factor (FGF-2), and increased expression of Ngb that remains upregulated until the sixth day post-injury (72). In this respect, other studies have demonstrated an increased expression of Ngb with stroke, hypoxia, and ischemia (73, 74). In an experimental model of TBI, overexpression of Ngb correlated with a significant reduction in sensorimotor deficits compared with a control group that did not overexpressed Ngb. The immunohistochemical analysis of injured cortex and hippocampus revealed that Ngb is mainly expressed in neurons and glial cells (75).

Human studies have correlated the genetic polymorphisms of the Ngb with susceptibility to neurodegeneration. One of these studies showed that decreased expression of Ngb in the elderly is associated with an increased risk of AD (76). In a preclinical study using transgenic AD mice, it was found that intracerebroventricular injection of Ngb decreased the formation of Aβ peptides, and the mitochondrial dysfunction, apoptosis, and neuronal death in the AD brains. In addition, other studies suggested that the neuroprotective effects of Ngb involved the inhibition of caspase-3 and 9, the activation of the PI3K/Akt pathway (77), and the removal of proteins aggregates (78). Finally, other study has demonstrated that Ngb is related to the neurotoxic effect produced by CNS 1-bromopropane (1-BP), a volatile organic compound implicated in damage to both the ozone layer of the atmosphere and the CNS. This compound is used as cleaning agent for metal, electronics, and optical instruments, as well as a substrate for the synthesis of pharmaceuticals and insecticides (79). The frequent use of this compound may cause health problems in mammals. For example, a previous study showed that exposure to 1-BP resulted in cognitive deficits and increased levels of 4-HNE and MDA modified proteins in rats (80). Also, rats exposed to 1-BP showed elongated astrocytic processes, a decreased number of oligodendrocytes, suggesting possible negative effects on myelination and degeneration of granular and Purkinje cells in the cerebellum (81). Similarly, a previous study showed that occupational exposure to 1-BP resulted in CNS adverse effects and peripheral neuropathy (82). Some evidence suggests that Ngb dysfunction may be involved with the toxic effects of 1-BP on CNS. For example, 1-BP correlated in a dose-dependent manner with a decrease in Ngb, cognitive dysfunction in rats, and a significant loss of neurons in layer V of the prelimbic cortex. These results suggest that the decreased expression of Ngb probably plays an important role in CNS neurotoxicity induced by 1-BP (83).

It is important to highlight that the presence of Ngb *in vivo* or *in vitro* depends on specific astrocytes (84) and the brain region affected. For example, in one study, Ngb-positive astrocytes were mostly found in the rhinencephalon region severely damaged in terms of hemorrhage (85, 86). However, Ngb was not detected in astrocytes from healthy mouse brains, suggesting that Ngb may have cytoprotective properties with the potential to be a therapeutic agent for intervention. However, the potential neuroprotective effect of Ngb in astrocytes (28) during ischemia (84) has neither been characterized nor the role of Ngb in neurogenesis and glial scar (28). In addition, whether Ngb is secreted by astrocytes as a neuroprotective agent has not been explored, and it requires further investigations.

## Neuroprotective Potential of Neuroglobin

Until recently, hemoglobin (Hb) was the most studied member of the globin family of proteins because of its oxygen-binding affinity in blood. Nevertheless, recent studies have revealed expression of other globin proteins in erythrocytes of vertebrates, including myoglobin (Mb), cytoglobin (CYGB), globin E (GbE), globin Y (GBY), and Ngb (87). Ngb is a 17-kDa monomeric protein, which shows a classic folded structure adapted to hold the heme-hexa-Fe-type HisF8 HisE7 in both the ferric and ferrous forms (26). According to homology studies, it was found that Ngb sequence is highly conserved between species, accounting for almost 76% of sequence conservation between humans and amphibians (88). Ngb is not only expressed in the nervous system (22) but also in the eyes, intestine, and ovary; however, no expression has been detected in kidney liver, heart, and skeletal muscle (89). In the brain, Ngb has been found in different regions including the cortex, thalamus, cerebellum, hippocampus, and hypothalamus (90). These areas are important in the processing of sensations, memory, and learning, and are often affected in hypoxic and ischemic insult or traumatic injuries.

Ngb, with its molecular properties, has been characterized as a protein responsible for O_2_ transport and scavenging of ROS and as O_2_-sensor and oxygen transporter (91). These functions suggest that the presence of Ngb is a key factor to brain homeostasis. At present, there are several studies addressing the role of Ngb in different pathologies such as focal ischemia and hypoxic–ischemic injuries (92–95). These studies suggest that Ngb serves as a sensor to hypoxic stress and has a protective effect. For example, Tiso et al. (96) reported that a nitrite reductase activity of Ngb inhibited mitochondrial respiration in presence of nitrite *in vitro* and suppressed oxygen consumption and ROS production. Current studies have shown a direct functional relationship between mitochondrial integrity and Ngb *in vivo* (74). For example, Ngb is associated with reduced oxidative damage induced by either reactive nitrogen species (RNS) or ROS (97). Moreover, Ngb structure was found to be extremely stable, in which its holoprotein was able to support temperatures exceeding 100°C and low pH values of up to 2.0 before denaturation (98). Finally, this protein may be involved in enhancing G-protein signal transduction by inhibiting the dissociation of guanosine diphosphate from the G-α subunit (73, 99, 100) and therefore involved in cell signaling processes (66).

Initially, Ngb was considered to be exclusively a cytoplasmic protein (101), but recent confocal microscopy studies have shown that it is also located in mitochondria and nucleus (102, 103). In this respect, it was shown that Ngb is associated with microdomains of lipid rafts and α-subunits of heterotrimeric protein G and becomes activated during oxidative stress, undergoing structural changes that lead to neuroprotection (103). On the other hand, the mitochondrial expression of Ngb becomes increased after oxygen-glucose deprivation (OGD) in primary cultures of mouse cortical neurons (104, 105). Furthermore, mitochondrial Ngb has interactions with cytochrome *c* and the voltage-dependent anion channel (VDAC), suggesting the importance of Ngb in mitochondrial function and neuroprotection (73, 106, 107). Additionally, it has been shown the influence of thyroid hormones on Ngb expression (97, 108). In one of these studies, the authors evaluated Ngb expression in different areas of the rat brain after T3 (100 L/100 g) administration. The authors found that T3 increased Ngb expression in the hippocampus and cerebellum; however, in cerebellum, Ngb expression was only detected at 120 min, 6 h, 12 h, and 24 h after T3 injection (97).

Other studies have shown that Ngb promoter is regulated by the transcription factors NFκB, SP1, and CREB (109), the hypoxia-inducible factor 1-α (HIF-1α), erythropoietin (EPO), and the VEGF (84, 110). In this respect, it was observed that VEGF2 increased the expression of Ngb by stimulating the Flk1 receptor, which in turn induced the expression of HIF-α (111).

The mechanisms of neuroprotection by Ngb have not been completely elucidated. Various stimuli can affect the expression of Ngb in different tissues, including the CNS. For example, in the cardiac tissue, which is also affected by ischemia, oxidative stress, and reperfusion, Ngb has been involved in the protection against cardiac hypertrophy induced by oxidation in cardiomyocytes, preventing them from cell death by ROS and therefore can be a clinical candidate for the treatment of heart diseases (112). On the other hand, it has been shown that cochlear oxidative stress is the main cause of sensorineural hearing loss and for which so far there is no treatment. In this aspect, Ngb is highly expressed in the cochlear nuclei and the superior olivary complex (SOC). Moreover, it was reported that Ngb is colocalized with the antioxidant neuronal protein nitric oxide synthase (NOS) in the SOC, suggesting the importance of Ngb in oxygen homeostasis and energetic metabolism in the auditory nervous system (113) (see Table 1). Ngb has been also involved in calcium homeostasis (27), ATP storage, inhibition of actin assembly, and response against increased hydrogen peroxide ion levels_._ This evidence suggests that Ngb is also involved in the maintenance of the integrity of the cytoskeleton, cell viability, neuroprotection, and glutamate removal (114).

**Table 1:** **Description of the fundamental aspects and biological effects of neuroglobin**.

Aspect	Description	Reference
Expression of neuroglobin in the CNS	Cerebellum and hippocampus	(97)
	Cortex, thalamus, cerebellum, hippocampus, and hypothalamus	(90)
Non-neuronal cells expressing neuroglobin	Cardiomyocytes	(112)
	Spiral ganglion cells and the superior olivary complex stem auditory	(113)
	Retina cells	(115)
Antioxidant role of neuroglobin	Regular removal of nitric oxide	(26, 116)
	Reduce the damage induced by reactive nitrogen species	(97)
Antiapoptotic role of neuroglobin	Survival in nerve cells overexpressing neuroglobin	(24)
	Decrease apoptosome formation	(25)
	Cytochrome *c* reduction	(24)
	Decreased levels of calcium – upholding levels of ATP – mitochondrial membrane potential in cultured neuronal cells	(27, 117)
	Modulation of metals such as iron, copper, and zinc in cultured neuronal cells	(117)
	Increased ATP reservoirs in cultured human neuronal cells	(114)
Signaling pathways involving neuroglobin	Inhibits the dissociation of guanosine diphosphate from protein G-α	(99, 100)
	It binds to the subunit Gβχ that activates PI3K and Akt in cultured human neuronal cells	(114)
	Inhibits production of IP3	(118, 119)
	Inhibits actin assembly-mediated Rac-1 in neurons	(120)
Factors that mediate expression of neuroglobin	HIF-1α	(110)
	NFκB–SP1–CREB	(109)
	VEGF	(84, 111)
	EPO	(84)
Drugs that increase neuroglobin expression in neurons	Deferoxamine–valproic acid–cinnamic acid in HN33 (mouse hippocampal neuron × N18TG2 neuroblastoma) cells	(121)
Neuroglobin expression in astrocytes	Neuroglobin after a subacute and chronic traumatic brain injury	(28)
	Neuroglobin in microglia and astrocytes after traumatic brain injury	(84, 122)
	Neuroglobin astrocytes through estrogen receptor ERβ	(22)
	Co-localization of neuroglobin with GFAP in human brain after a stroke	(28)
	Neuroglobin protection is mediated *via* Raf/MEK/ERK through 14-3-3r	(123)
	Neuroglobin expression is dependent on the activation of estrogen receptor beta; tibolone induces the upregulation of Ngb	(65)
	Testosterone upregulates Ngb expression in glucose deprived cells	(124)
Related pathologies involving neuroglobin	Cerebral hypoxia	(70, 71, 125, 126)
	Focal cerebral ischemia	(127)
	Alzheimer	(76)
	Injury in the cerebral cortex	(72)
	Stroke	(73)
	Traumatic brain injury	(74)
	Removal of proteins capable of forming aggregates deleterious	(78)
	Neurotoxic effect of 1-bromopropane	(83)

Recent studies have used different drugs to increase Ngb expression in neurons as a therapeutic neuroprotective agent in traumatic brain injury. Among the drugs studied are deferoxamine, an iron chelator, valproic acid, and cinnamic acid (121). Further *in vivo* studies are needed in order to determine both the induction levels of Ngb by these drugs and also if they have adverse effects in the sensorimotor or cognitive recovery after traumatic brain injury or brain ischemic injury.

### Antioxidant Effect

Ngb has very little affinity for oxygen, and the oxygenated species formed with Ngb are unstable; thus, it does not provide a stable source of oxygen, due to the low concentration of Ngb in neurons (128). In fact, it is recognized that one of the roles of Ngb is basically its affinity for NO (26, 129), and this action can be related to the clearance of this gaseous ligand. Moreover, Ngb has been shown to act as a ROS and RNS scavenger in different models (84, 112, 122), although its antioxidant activity is lower than that of *N*-acetyl cysteine, glutathione, and vitamin C (115). Finally, Ngb has interactions with many antioxidant-related proteins such as Cyt *c*, Thio, AIF, Prdx3, 4, and 6, Thop1, and Dj1, among others. However, further research is needed in order to address the relevance of the antioxidant effects of Ngb in CNS diseases.

### Antiapoptotic Effect of Ngb

The process of apoptosis is complex, and few have investigated the action of Ngb. However, some studies used computational modeling to determine the mechanism of Ngb in cell death. For example, data obtained using computational modeling suggest that Ngb reduces the formation of the apoptosome by a redox reaction with cytochrome *c* (24), causing the blocking of initiator pro-caspase 9 activation and thus significantly blocking the triggering of apoptosis. In the same study, the authors simulated the interaction between Ngb and cytochrome *c* and validated their results using an *in vitro* approach. The authors found a very rapid reaction between reduced (ferrous) neuroglobin and oxidized (ferric) cytochrome *c* (24), suggesting that Ngb might affect the initiation of apoptosis by interacting with cytochrome *c*. Interestingly, under normal conditions, these molecules do not interact with each other, but under stress, Ngb prevents cytochrome *c* release from mitochondria, thus protecting the cell from apoptosis (115). In this respect, it was reported that after reducing cytochrome *c*, Ngb, in the ferric form, binds to two receptors coupled to G protein subunits (GPCR). This fact is interesting, as the G-α subunit can cause an inhibition in the production of IP3 (inosine triphosphate) and reduce cytosolic calcium release (118, 119). On the other hand, Ngb binds to the Gβχ subunit, activating PI3K and Akt and thus promoting cell viability (114). Also, as mentioned before, Ngb inhibits Rac-1, Pak1 kinase, and actin assembly, preventing cytoskeletal rearrangement and avoiding the initiation of death signaling (120). Moreover, Ngb expression has been found to be higher in metabolically active cells, such as neurons and retinal cells, which have some features in common such as high cytosolic calcium levels, which can trigger apoptosis (115). This aspect has already been validated experimentally, showing that increased Ngb expression is directly linked to calcium homeostasis and maintenance of both mitochondrial membrane potential and ATP levels (27). Ngb may be also important in the modulation of metallic ions such as Fe, Cu, and Zn, which are increased in neurons under hypoxic conditions. These ions can induce inflammation, mitochondrial damage, ROS production, and the release of neurotransmitters, leading to neuronal death excitotoxicity (117). All these findings support the role of Ngb in apoptotic regulation, a subject that merits further research.

## Neuroglobin Expression in Astrocytes

The expression of Ngb is evident in neurons and the protective role of Ngb in neuronal cells has been well documented; however, the function of Ngb in astrocytes is less well studied (84). In 2000, a study reinforced the expression of Ngb mRNA in spinal cord funicles, hippocampal alveus, and cerebellar medulla of rodents (101). Until recently, only few studies have reported the expression and function of Ngb in astrocytes (28, 84, 122, 130). Previously, in 2005, a study reported that Ngb mRNA was detected in primary cultures of cortical astrocytes and transfection of these astrocytes with anti-sense for Ngb led to a 2.5-fold increase in apoptotic cells when compared to controls, suggesting a possible protective role of Ngb expression in astrocytes against insult (131).

Consistently, Avivi et al. reported that the subterranean mole rat (Spalax) expresses Ngb in neurons and astrocytes isolated from the corpus callosum (125). More recently, Lechauve et al. reported that Ngb was detected in astrocytes processes optic nerve under physiological conditions *in vivo* (132). Indeed, it is important to mention that Ngb expression has been also observed in astrocytes under pathological conditions (e.g., reactive astrocytes). For example, the expression of Ngb was found upregulated in reactive astrocytes located in the proximity of a penetrating cortical injury *in vivo* (22, 133), in Müller cells during reactive gliosis (132) or located in regions associated with the most severe pathology and the astroglial scar in murine models (134). Moreover, Ngb was also detected in astrocytic tumors such as rat astrocytoma cells (C6) and human astrocytoma cells (U251) (135, 136), thus confirming the existence of Ngb in tumoral cell lines.

**Figure 1:**
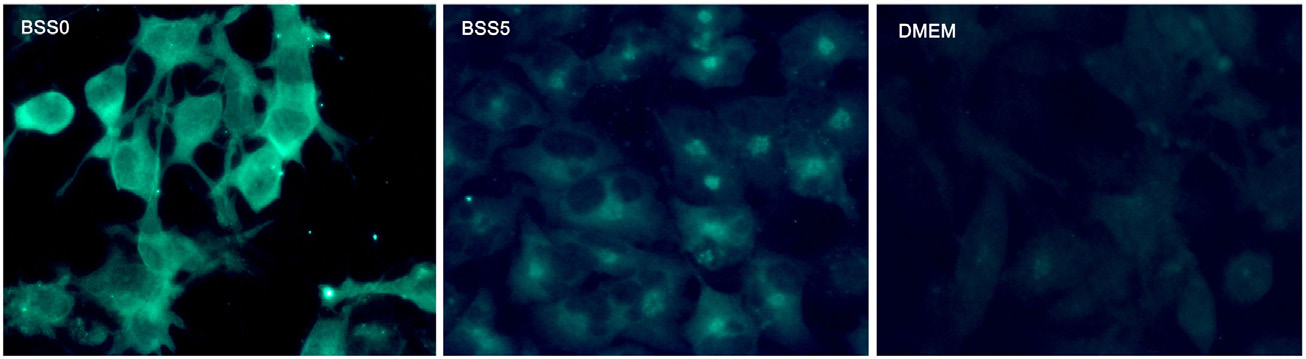
**Representative microphotographs of astrocyte-like cells (T98G cell line) expressing neuroglobin**. Data generated in our group showed that in cells subjected to metabolic insult by adding a balanced salt solution devoid of glucose (BSS0), neuroglobin expression is enhanced and homogeneously distributed in the cytoplasm (left). The control condition (BSS5) was the same as BSS0, but adding 5-mM glucose; in this case, neuroglobin expression was decreased in comparison with BSS0 and located in proximity of the cell nucleus (center). Under basal culture conditions with DMEM medium, Ngb expression was similar to that of BSS5.

A previous study evaluated Ngb expression in astrocytes after brain trauma and reported that Ngb expression is present in subacute and chronic injuries but not acute trauma (28). Moreover, in other studies, it was found that Ngb is expressed in microglia and astrocytes specifically during conditions such as traumatic brain injury (84, 122) and that estradiol regulates the expression of Ngb in astrocytes (66) through the estrogen receptor β (ERβ) (22), while ERα is involved in the regulation of Ngb in neurons (137). Interestingly, tibolone, a synthetic hormone with estrogenic, progestogenic, and androgenic actions, has also been reported to induce Ngb expression in astrocytic-like cells *in vitro* under glucose deprivation (65) (Figure 1). This expression was dependent on the activation of ERβ. Moreover, inhibition of Ngb by siRNA significantly affected the protective effects of tibolone in glucose-deprived cells, suggesting that its actions might be mediated by ERβ and Ngb upregulation (65). Similarly, testosterone also induced the expression of Ngb in astrocytes subjected to glucose deprivation, indicating that estrogenic and androgenic compounds might play a protective role *via* induction of Ngb (124). Furthermore, a previous report (130) showed co-localization of Ngb and GFAP in glia from human brains after stroke (28). It has been postulated that neuroprotection by Ngb in astrocytes is mediated by Raf/MEK/ERK pathway through 14-3-3r, which has the ability to bind to multiple signaling proteins as kinases, phosphatases, or transmembrane receptors (123). Controversially, Ngb has not been detected in astrocytes by conventional immunohistochemistry or fluorescent immunostaining in normal mice brains (134). Indeed, a strong correlation between the cellular expression of Ngb and the neuronal marker NeuN, but not the astroglial marker GFAP, has been found (130). These results indicate that is debatable whether Ngb is expressed in astrocytes and others glial cells under non-pathological conditions (84). Nevertheless, Ngb is detected in hypoxic mice brains (138), brain tissues in stroke patients, and specialized glial cells such as pituicytes (101). Therefore, it is clear that further studies are necessary in order to determine the role of Ngb in astrocyte-mediated neuroprotection.

## Conclusion and Perspectives

The studies reviewed here show that Ngb is a molecule with antioxidant and antiapoptotic properties acting on mitochondrial and cytosol mechanism of pathology. Therefore, Ngb can be considered as a potential target to decrease neural damage, and its enhanced expression after brain injury probably reflects endogenous mechanisms of neuroprotection. Ngb is upregulated by pharmacological compounds, such as estrogenic molecules. Overexpressing Ngb by gene therapy or its pharmacological induction may represent a potential therapeutic approach for the treatment of traumatic brain injury.

## Author Contributions

EB, GEB, VE, and LG-S wrote the manuscript; RC revised the manuscript; and MA-R provided the figure.

## Conflict of Interest Statement

The authors declare that the research was conducted in the absence of any commercial or financial relationships that could be construed as a potential conflict of interest.
